# A rapid qualitative analysis of health care worker and community focus groups on determinants and implementation strategies for long-acting PrEP in antenatal and postpartum settings in Kenya

**DOI:** 10.1007/s44250-026-00415-x

**Published:** 2026-08-26

**Authors:** Tessa Concepcion, Marin Strong, John Kinuthia, Felix A. Otieno, Eunita Akim, Helen Aketch, Laurén Gómez, Grace John-Stewart, Bih Moki Suh, Nancy Ngumbau, Emmaculate M. Nzove, Jerusha N. Mogaka, Sarah Obatsa, Ben O. Odhiambo, Caroline Omom, Anjuli D. Wagner, Salphine Watoyi, Jillian Pintye

**Affiliations:** 1 Department of Global Health, University of Washington, Seattle, USA; 2 School of Nursing, University of Washington, Seattle, USA; 3 Research and Programs Department, Kenyatta National Hospital, Nairobi, Kenya; 4 Department of Epidemiology, University of Washington, Seattle, USA; 5 Department of Medicine, University of Washington, Seattle, USA; 6 Department of Pediatrics, University of Washington, Seattle, USA; 7 Department of Biobehavioral Nursing and Health Informatics, University of Washington, Seattle, USA

**Keywords:** Long-acting PrEP, Maternal and child health, Pregnancy, Postpartum, Health system, Implementation determinants, Implementation strategies

## Abstract

**Background:**

New long-acting (LA-) PrEP modalities have demonstrated efficacy in HIV prevention and growing evidence of safety during pregnancy and lactation. There are unique considerations for LA-PrEP implementation in reproductive health settings, yet scarce implementation data are available. We identified potential barriers and facilitators and strategies for LA-PrEP implementation for pregnant and postpartum women.

**Methods:**

We conducted a qualitative study using focus group discussions (FGDs) with healthcare workers (HCW) at five clinics and community advisory board (CAB) members related to PrEP delivery for pregnant and postpartum women. Interview guides were informed by the EPIS and CFIR frameworks. HCW FGDs included an activity to prioritize information, training, or support strategies for LA-PrEP provision. We used a rapid qualitative analysis to identify facilitators and barriers to integrating LA-PrEP into reproductive health settings and matched potential implementation strategies.

**Results:**

A total of 9 FGDs were conducted with 45 individuals between August and October 2023 (5 FGDs among HCWs and 4 among CAB members). They identified interconnected determinants including training, funding, personnel, and community sensitization. Some strategies clearly addressed barriers (e.g. ongoing training to target access to knowledge). However, some barriers had no clear solution, such as LA-PrEP drug availability and access to funds to conduct training and community sensitization. The prioritization activity revealed common resources needed for LA-PrEP implementation (e.g., national guidelines, training, and personnel), but importance varied by location. A wide range of implementation strategies were identified, such as financial incentives for transportation, using community health providers to conduct community sensitization, and integrating LA-PrEP into family planning clinics.

**Conclusions:**

HCW and CAB members were optimistic about the promise of new LA-PrEP options and identified important barriers and strategies for consideration. Future research should consider prioritizing implementation strategies and evaluating the impact of strategies on implementation barriers. This study emphasizes the importance and potential impact of offering LA-PrEP options to pregnant and postpartum women.

**Supplementary Information:**

The online version contains supplementary material available at 10.1007/s44250-026-00415-x.

## Background

In high HIV burden settings, the incidence of HIV among pregnant and postpartum women is nearly double that of the general population [1, 2]. The World Health Organization (WHO) recommends oral pre-exposure prophylaxis (PrEP) for pregnant and postpartum women in high HIV-incidence settings, yet despite the proven safety and efficacy of oral PrEP for pregnant and breastfeeding women and their infants, its uptake remains low [3–8]. Integration of oral PrEP into reproductive health settings – such as maternal child health (MCH) clinics, antenatal care (ANC) clinics, and family planning (FP) clinics – improves uptake, service delivery, and client satisfaction [9–11]. However, studies also identified considerable barriers to integration, including workforce time burdens [11], staff shortages [12], insufficient PrEP training, limited privacy for delivering PrEP, and a lack of systematic scale up [13].

New long-acting (LA-) PrEP modalities, such as every two-month cabotegravir (CAB-LA), twice-yearly lenacapavir (LEN), and the monthly dapivirine vaginal ring (DVR), effectively prevent HIV, with growing evidence supporting their safety for pregnant and lactating populations [14–20]. Ensuring high-quality PrEP delivery in pregnancy and postpartum requires understanding the role of the community, assessing the impact of PrEP integration on workload and client volume, examining provider attitudes and beliefs, and ensuring provider training with clinical delivery, yet scarce implementation data exists to address the unique considerations of this context [21]. As countries work towards regulatory approval and introduction of new LA-PrEP formulations, early implementation studies can help narrow the research-to-practice gap [22]. Specifically, we aimed to identify potential facility-level barriers and facilitators for adoption and uptake of LA-PrEP for pregnant and postpartum women in Kenya, and to identify strategies to for implementation.

## Methods

### Study design

We conducted an exploratory qualitative study using content analysis with healthcare workers and other key opinion leaders related to PrEP delivery for pregnant and postpartum women. Focus group discussions (FGDs) were conducted as part of a larger clinical trial (NCT04472884) of PrEP adherence for pregnant and postpartum women [23]. Facility selection criteria, facility characteristics, and participant recruitment procedures have been described previously [23]. Briefly, these facilities are located in Kisumu and Siaya counties in Kenya, in a region with adult HIV prevalence of 19.9% (up to 28% among pregnant women) [24, 25]. At these sites, oral PrEP is provided as part of the standard package of HIV prevention services routinely offered to eligible clients accessing inpatient and outpatient services, including MCH clinics [25]. Focus groups, rather than individual interviews, were used to facilitate interaction among participants and elicit shared perspectives on PrEP implementation. A section of the interview guides was devoted to questions about LA-PrEP implementation. FGDs were held with two groups: healthcare workers (HCWs) at participating clinics and the study’s community advisory board (CAB). The study’s CAB was comprised of local community leaders, women from the community, the Kenyan Ministry of Health, and county health representatives from MCH departments. Focus groups were limited to a maximum of five participants to encourage a dynamic exchange of diverse perspectives while ensuring that each participant had the opportunity to contribute meaningfully to the discussion. HCWs were purposively sampled to include perspectives from different areas related to ANC-PrEP delivery at all five study sites, including providers involved in antenatal care, postnatal care, and PrEP service delivery (e.g., nurses, clinical officers, counselors, pharmacy technicians, mentor mothers, and facility leadership). To minimize perceived coercion, recruitment and consent were conducted by study staff not involved in clinical supervision, participation was voluntary, and it was emphasized that responses would not affect employment or standing within the clinic. All members from the parent study’s CAB were invited to participate.

### Data collection

Interviewers (SO, HA, CO) attended a 3-day training session in July of 2023 and internally piloted materials. They were from the same region as participants, were female, held a bachelor’s degree, and have prior training and current employment in qualitative interviewing. All interviewers spoke English as well as Swahili and/or Luo (local language). Interviewers used a semi-structured interview guide with a section devoted to themes around LA-PrEP acceptability, informed by the Exploration, Preparation, Implementation, Sustainment (EPIS) framework [26] and updated Consolidated Framework for Implementation Research (CFIR) [27] with additional domains and constructs added for use in LMICs [28]. More specifically, the questions were informed by the Exploration and Preparation domains of EPIS because LA-PrEP, as an evidence-based intervention, had been established at the time of the FGDs but had not yet been implemented in the Western region of Kenya, where the study is situated. Similarly, not all CFIR constructs were applicable, and the selected constructs that informed the guide have been mapped to questions and are available for review in the Appendix. These frameworks provide complementary, well-established structures for examining multilevel determinants of implementation and acceptability across different stages, with adaptations that enhance relevance to low-resource contexts. A study staff member familiar with the selected participants contacted them by phone and introduced them to the interviewer, who then invited them to participate in an FGD. Prior to the FGD, participants were informed of current LA-PrEP methods, including CAB-LA and DVR, which at the time were the closest to implementation and being evaluated for safety in pregnant women. Each focus group had a facilitator who led the discussion and a note-taker who kept detailed notes to support transcription and FGD debriefs. All FGDs were conducted in person in a private room with no one else present. FGDs were conducted in the participants preferred language (English, Luo, or Swahili) and lasted approximately 90–120 min. No repeat or follow-up interviews were conducted.

HCW groups also engaged in a prioritization activity. In the prioritization activity, the facilitator asked “What information, training, or support would you need to feel comfortable offering a long-acting form of PrEP to pregnant and postpartum women?” The note taker recorded all suggested strategies on a large poster. The group then ranked all suggested strategies first, from easiest to hardest to implement (“Which of your suggested ideas would be the easiest to implement or do in your clinic?”) and second, from most to least helpful (“Which of your suggested ideas would be the most helpful to make you feel comfortable offering a long-acting PrEP in your clinic?”). HCWs were not restricted to selecting the same strategies across the two prioritization questions and were not limited to a fixed number of strategies in order to capture a broader range of context-specific priorities and perspectives. Within each prioritization question and subsequent ranking, the facilitator encouraged the group to reach a consensus.

### Data management and analysis

FGDs were audio-recorded, transcribed, and translated into English (SO, HA, CO). Interviewers completed debrief reports within 24 h of completing an FGD. Weekly meetings were held to ensure protocol fidelity. One HCW and one CAB FGD were back-translated for quality assurance. Back-translation confirmed high translation fidelity, with no substantive discrepancies identified and no resulting changes to study procedures. Two authors reviewed transcripts for accuracy (EA, JN). We used a rapid qualitative analysis approach (RQA) to identify facilitators and barriers to integrating LA-PrEP into antenatal care settings and potential implementation strategies needed to ensure successful implementation [29–31]. This approach was chosen based on our structured interview guide, to streamline data synthesis, and to enable timely responses to our research objective, allowing for swift identification of determinants and strategies while preserving data richness and rigor. First, authors (TC and MS) created a summary sheet template deductively based on the semi-structured interview guides. The template included brief domain names corresponding to interview guide questions. We piloted the template with two transcripts to ensure reliability across researchers. Summary sheets included sections for brief quotes or transcript line numbers allowing authors to re-examine original data as needed for clarification, validation, and expansion. Second, transcripts were divided between researchers, and each researcher summarized interview findings into major summary points according to the summary sheet domains. Authors (TC and MS) reviewed summary sheets together and resolved discrepancies through discussion and consensus. Third, we developed matrices to organize themes, whereby rows were individual FGDs and columns were summary sheet domains. Matrices were developed in Microsoft Word (Microsoft^®^ Word for Microsoft 365 MSO, Version 2409) and reviewed by the lead author (TC). Fourth, we extracted themes by reviewing matrix data within each column. Fifth, we coded summary points for CFIR determinants within the matrices using Microsoft Excel (Microsoft^®^ Excel^®^ for Microsoft 365 MSO, Version 2501). Sixth, we concurrently mapped summary points to participant-identified implementation strategies, labeled and organized per the Expert Recommendations for Implementing Change (ERIC) implementation strategies. Implementation barriers and strategies were summarized by domain, and FGD transcripts were reviewed again to identify any missing items. The consolidated criteria for reporting qualitative studies (COREQ) and Planning for and Assessing Rigor in Rapid Qualitative Analysis (PARRQA) were used as a guide throughout data collection and analysis [32, 33].

### Ethical considerations

The University of Washington and Kenyatta National Hospital institutional review boards approved this study. All participants provided electronic written informed consent before study participation and consented to be audio recorded.

## Results

A total of 9 FGDs were conducted among 45 individuals between August and October 2023 (5 FGDs among HCWs and 4 among CAB members) with five participants in each FGD. One HCW was unable to join due to a work conflict, so a replacement with a similar role was invited. Both HCW and CAB participants were predominantly female (72% of HCW and 60% of CAB) (Table 1). Median age was 35 years for HCW (range: 28–63) and 43 years for CAB members (range: 23–70). Most FGD participants had a university/college education (92% of HCW and 75% of CAB).

**Table 1:** HCW and CAB demographics

	HCW, *N* = 25		CAB, *N* = 20
**Gender**			
Female	18 (72%)		12 (60%)
Male	7 (28%)		8 (40%)
**Age** *median (range)*	35 (28–63)		43 (23–70)
Missing			1
**Highest education achieved**			
Secondary	2 (8.0%)		4 (20%)
Polytechnic	0 (0%)		1 (5.0%)
University/college	23 (92%)		15 (75%)
**Work location**		**Role on CAB**	
MCH clinics	14 (56%)	PrEP ambassador	5 (25%)
CCC	9 (36%)	County government leader	4 (20%)
FP clinic	1 (4.0%)	Community leader	4 (20%)
MCH and FP clinic	1 (4.0%)	Healthcare planner	4 (20%)
		Health system administrator	3 (15%)
		Community health volunteer	2 (10%)
		Youth representatives	1 (5.0%)
**Employment**		**Employment**	
Counselor	9 (36%)	Other health services	6 (30%)
Nurse	7 (28%)	Administrative role	5 (25%)
Pharmacy technician	3 (12%)	Nurse	3 (15%)
Clinical officer	2 (8.0%)	Youth/community outreach	2 (10%)
Mentor mother	2 (8.0%)	Unemployed	2 (10%)
Department in-charge	1 (4.0%)	Civil/public service	1 (5.0%)
Other clinician	1 (4.0%)	Education	1 (5.0%)
**Number of years…** *median (range)*			
In current role	8 (4–38)		
Working on issues related to women, pregnancy, and HIV prevention	8 (2–23)		
Experience caring for pregnant or postpartum women	6 (3–25)		
Providing PrEP to pregnant or postpartum women	4 (1–12)		
**Received specific training in…**			
providing PrEP to pregnant or postpartum women	15 (60%)		
PrEP adherence counseling for pregnant or postpartum women	14 (56%)		

MCH: Maternal and child health; CCC: Comprehensive care clinic; FP: family planning

### Cross-cutting implementation determinants of LA-PrEP

**Fig. 1:**
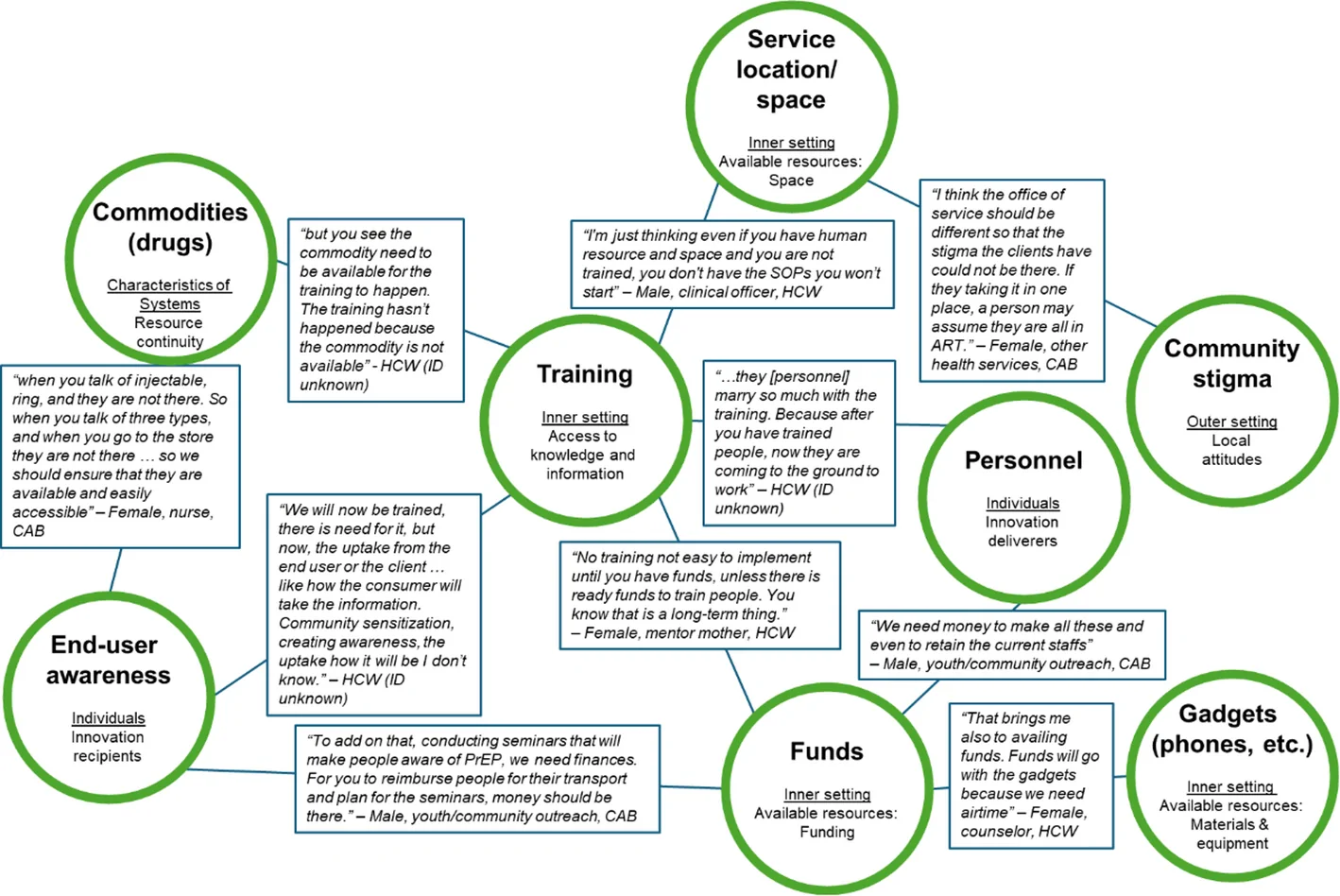
Connections made by HCW and CAB members between LA-PrEP implementation determinants (green circles, with associated CFIR construct)

Grounded in the CFIR, we identified HCW perceptions of important determinants influencing implementation determinants for future integration of LA-PrEP into reproductive health settings such as MCH clinics, ANC clinics, and FP clinics. FGDs explicitly identified determinants that were interconnected and dependent on one another (Fig. 1). Training emerged as a central determinant, with links to other factors such as funding, personnel, service location, and community stigma. Participants noted that training cannot occur effectively without the availability of necessary commodities, such as injectable PrEP or rings, emphasizing the importance of ensuring that these products are accessible. Similarly, financial resources were identified as essential to support training activities, reimburse participants for transport, and ensure the availability of required materials, with one participant highlighting that funding gaps can lead to delays in implementation.

*“We need money to make all these [things happen] and even to retain the current staffs.” – male*,* youth/community outreach*,* CAB*

Personnel were also highlighted as a key determinant for LA-PrEP implementation, with participants emphasizing the need to retain staff through both training and financial incentives. FGD participants identified many implementation barriers and facilitators to implementing LA-PrEP in their settings. CFIR constructs and supporting quotations can be found in Table 2.

The interconnected determinants described above provide the context for understanding specific barriers and facilitators to LA-PrEP implementation (Fig. 1). Below, we present findings organized by CFIR domain, highlighting the constructs that participants identified as most influential for successful integration of LA-PrEP into reproductive health services.

**Table 2:** Implementation determinants for integration of LA-PrEP into reproductive healthcare settings, organized by CFIR constructs

CFIR CONSTRUCT	SUPPORTING QUOTATION
**Intervention characteristics**	
Relative advantage	*“Let me talk about the pregnant women*,* I feel the other two options [injection and ring] will be better because*,* you know in pregnancy you have IFAS [iron and folic acid supplements] pill burden. Maybe you are sick*,* you have malaria. Then there is also this aspect of the first trimester*,* nausea and vomiting. The drugs cannot be accommodative. So you know if we give the injection is a better option because it is one off thing*,* or the ring is just inserted and is like that. So I feel talking on the basis of a pregnant woman that will favor them.” – Female*,* counselor*,* HCW*
Cost	*“if there is cost implication then it should be well indicated clearly” – Female*,* healthcare planner*,* CAB*
	*“When we talk about vulnerability*,* someone who has constrain in finances will find it difficult to get drugs. That is moving from home to the facility to get the drugs.” – Male*,* community leader*,* CAB*
**Outer setting**	
Local attitudes	*“It [long-acting PrEP] will increase unfaithfulness because you will not be walking with the drugs [oral PrEP] with you every day” – Female*,* PrEP ambassador*,* CAB*
	*“Some of them [PrEP users] lie. So they should be asked*,* and then they say which one do you opt for? They will tell you that*,* ‘I don’t want that tablet; I have already been injected elsewhere.’ You see*,* so those are some of the challenges we can expect.” – Female*,* county government leader*,* CAB*
Local conditions	*“I would try to bring in the issue of environment*,* environmental factors that are sometimes beyond the control of the leaders*,* the community and even the government. For example*,* when there is very harsh environmental impact like El Nino*,* you may have the information*,* you may have your willing community*,* you may have willing leadership*,* but how to get to that place during that harsh environmental condition is a problem*,* So sometimes environment plays a factor and even hinders physical or even communication access of information to the community.” Male*,* health system administrator*,* CAB*
Partnerships & connections	*“It needs all heads to come together from the partner*,* national government*,* county government and the health providers. If they can come together for this to be rolled down.” – Male*,* community health volunteer*,* CAB*
Policies & laws	*“They [the government] are also the ones that make policies that PrEP is safe for the public.” – Male*,* community health volunteer*,* CAB*
	*“And you know guidelines are not within our [scope]… it is national guideline.” – Female*,* mentor mother*,* HCW*
Policies & laws, community characteristics	*“People tend to believe that anything which is not signed by the government is null and void. If someone is taking samples of blood and the chief doesn’t know*,* then no one will partake in it.” – Male*,* community leader*,* CAB*
**Inner setting**	
Structural characteristics: Physical infrastructure, work infrastructure	*“Because of the setup which we have that [family planning department] is the only available space… I think structurally we have maybe rooms to implement and we also have*,* though limited*,* we have trained [personnel] and I am very sure they will still be trained.” – Male*,* counselor*,* HCW*
Compatibility	*“The same work I am going to do for someone who has come for oral PrEP or the injectable PrEP*,* it is the same work.” – Female*,* nurse*,* HCW*
	*“On the same note*,* I feel it will reduce our workload because we shall be giving these injections for two months*,* this mother stays at home for two months then she comes back*,* unlike the orals whereby you keep on reminding them through the message.” – Male*,* clinical officer*,* HCW*
	*“Another challenge is the workload. I am in family clinic*,* and I give a lot of services. You will find me doing antenatal checkup for pregnant mothers*,* I do cancer screening*,* and I have to give the information about PrEP. So*,* at times*,* I feel so overwhelmed.” – Female*,* nurse*,* HCW*
	*“For example*,* the way we do it in family planning*,* you put everything on the table*,* then you discuss one by one so that you allow the client to make an informed choice. They can do it the same way [for] PrEP.” – Female*,* healthcare planner*,* CAB*
Incentive systems	*“Proper training for the people who are going to carry this exercise*,* give them enough knowledge because there is a new thing in the market*,* and motivating them because we want it to work well” – Male*,* healthcare planner*,* CAB*
Relative priority	*“How do we integrate? What about the partners who are pulling out*,* who will continue with these services? So they [government] choose people [select HCW] to take to seminars or to the training” – Male*,* healthcare planner*,* CAB*
Available resources: Funds	*“No training*,* not easy to implement until you have funds*,* unless there is ready funds to train people. You know that is a long-term thing.” – Female*,* mentor mother*,* HCW*
Available resources: Space Access to knowledge and information	*“I’m just thinking even if you have human resource and space and you are not trained*,* you don’t have the SOPs*,* you won’t start.” – Male*,* nurse*,* HCW*
Access to knowledge and information	*“We still don’t have sufficient data*,* we don’t want to get into a situation like the one that we found our self during the vaccination of Covid-19*,* then the client is there asking you the question before getting the jab*,* that ‘is it true that I will not give birth to children’.” HCW*,* ID unknown*
	*“I will comfortably be providing the information [about LA-PrEP] but still we need additional training to keep us more up to date. Things change here and there and us providers we need to be top of it all to give the correct information every time.” Male*,* pharmacy tech*,* HCW*
Collective efficacy	*“Yesterday we had a meeting*,* and they were complaining*,* ‘You people of the program*,* you do take your people for training*,* and you leave us behind. Now we can’t offer the services.’” – Female*,* counselor*,* HCW*
**Individuals**	
High-level leaders	*“in Kenya now we have something called government to government. We source our commodities from outside and truly depends with the relationship between our government and the other countries government where at one point you get difficult leadership the other side and they stop the flowing of the support then we don’t get commodities especially drugs*,* test kits and all that. So*,* government plays some major role in that they have to do government to government*,* and they must be good relationship so*,* that the other ideas*,* the other commodities flow in.” – Male*,* county government leader*,* CAB*
Innovation recipients	*“…Some people are not ready to look at what is down there*,* so it will depend on the individual—will you be ready to see and even touch what is down there?” – Female*,* healthcare planner*,* CAB*
	*“Currently we are administering [the ring] and a few concerns are some say the ring might disappear in the body and they might not get pregnant. Those are concerns that are being raised but we are trying to give relevant information*,* so that these clients can choose the best information.” – Male*,* nurse*,* HCW*
Implementation facilitators	*“through the CHPs [community health providers] in the community and even in the community meeting where HCW can be invited to give that information [about LA-PrEP] to different groups of people” – Female*,* healthcare planner*,* CAB*
Other implementation support	*“I think the best is the groupings say youth groups*,* we have that youth groups are a very good avenue for reaching the youths and then they can get the information.” – Male*,* county government leader*,* CAB*
**Implementation Process**	
Assessing needs: innovation recipients	*“You can also help the patient come up with [an option] that can work with them.” – Female*,* counselor*,* HCW*
**Characteristics of Systems**	
External funding agent priorities	*“even the partner when they come*,* they only concentrate on their particular staffs*,* so their trainings for their staffs everything that they are doing is that they are just concerned about their staff.” – Female*,* healthcare planner*,* CAB*
Resource source	*“to this facility*,* availability of the commodity [LA-PrEP]*,* according to the situation that we have now*,* if it is only left for the ministry*,* not donor*,* we may have a problem.” – Female*,* counselor*,* HCW*
**Characteristics of the Intervention**	
Perceived sustainability	*“…as of now*,* if the partners pull out*,* you’ll start from zero*,* because the information will be gone with the partners who were…the staffs who were working with the partners.” – Female*,* healthcare planner*,* CAB*

### Intervention characteristics

HCW and CAB participants largely agreed that injectable PrEP has significant advantages over daily oral PrEP due to its discreet use and convenient dosing schedule (CFIR construct: **relative advantage**). Some participants were aware of recent clinical trials and referenced the high effectiveness of bi-monthly and twice-yearly injectable PrEP (CFIR construct: **evidence-base**). However, they emphasized that pregnant and postpartum women would be particularly concerned about safety for their baby. Injectable PrEP was noted as simple to administer (CFIR construct: **complexity**) and could be integrated into clinics in a manner similar to family planning injections (CFIR construct: **design**). Concerns were raised about costs, including participant affordability (CFIR construct: **cost**).

*“When we talk about vulnerability*,* someone who has constrain in finances will find it difficult to get drugs. That is moving from home to the facility to get the drugs.” – Male*,* community leader*,* CAB*

### Outer setting

Despite broad agreement that multiple PrEP options would be beneficial for pregnant and postpartum women, CAB members noted that local attitudes could interpret the discretion of longer-acting options negatively, contributing to stigma (CFIR construct: **Local attitudes**). One CAB member noted that local conditions in more remote communities, such as limited access to information infrastructure and environmental disruptions such as extreme weather, may delay awareness and uptake of new LA-PrEP options (CFIR construct: **Local conditions**). Participants emphasized that successful implementation requires strong partnerships between government, health providers, and community leaders (CFIR construct: **partnerships & connections**). Local leaders, including village elders, clan elders, church leaders, community health volunteers, chiefs, and their assistants, were identified as important opinion leaders. Both CAB and HCW noted that national guidelines from the government were essential for LA-PrEP implementation (CFIR construct: **policies & laws**):

*“People tend to believe that anything which is not signed by the government is null and void. If someone is taking samples of blood and the chief doesn’t know*,* then no one will partake in it.” – Male*,* community leader*,* CAB*

### Inner setting

HCW and CAB members drew parallels between LA-PrEP and family planning implementation. Some HCWs believed it could be smoothly integrated into existing services, such as family planning, while others raised concerns about limited space and high workload (CFIR construct: **structural characteristics**). Participants had mixed opinions on how the addition of multiple PrEP options would impact workflow (CFIR construct: **compatibility**), some thought it would reduce workload due to less frequent dosing schedules and alignment with existing family planning procedures, while others highlighted that they are already overwhelmed, and demand might increase with an injectable option.

*“On the same note*,* I feel it will reduce our workload because we shall be giving these injections for two months*,* this mother stays at home for two months then she comes back*,* unlike the orals whereby you keep on reminding them through the message.” – Male*,* clinical officer*,* HCW*

*“Another challenge is the workload. I am in family clinic*,* and I give a lot of services. You will find me doing antenatal checkup for pregnant mothers*,* I do cancer screening*,* and I have to give the information about PrEP. So*,* at times*,* I feel so overwhelmed.” – Female*,* nurse*,* HCW*

HCWs expressed confidence that proper training would enable them to counsel on multiple PrEP options effectively (CFIR construct: **access to knowledge and information**). HCWs cautioned that insufficient data could lead to injection hesitancy, akin to what was observed with the COVID-19 vaccine. They noted that ongoing training, or refresher trainings, would be helpful to keep them up to date on new information and train new staff.

*“I will comfortably be providing the information [about LA-PrEP] but still we need additional training to keep us more up to date. Things change here and there and us providers we need to be top of it all to give the correct information every time.” Male*,* pharmacy tech*,* HCW*

FGD participants expressed concerns related to training, particularly that only few individuals within healthcare systems are trained and others are left without information (CFIR constructs: **relative priority**,** collective efficacy**). CAB specifically mentioned concerns around service continuity in the context of external implementing partners “pulling out”, which may reflect local grant closures and shifts in donor priorities.

*“How do we integrate? What about the partners who are pulling out*,* who will continue with these services? So they [government] choose people [select HCW] to take to seminars or to the training” – Male*,* healthcare planner*,* CAB*

*“They [other HCW] were complaining*,* ‘You people of the program*,* you do take your people for training*,* and you leave us behind. Now we can’t offer the services.’” – Female*,* counselor*,* HCW*

### Individuals

Participants emphasized the role of government leadership to ensure a stable supply of PrEP, noting that relationships with other countries can determine availability of PrEP at a clinic level (CFIR construct: **high-level leaders**). HCWs reported being comfortable counseling clients on multiple PrEP options, including LA-PrEP (CFIR construct: **innovation deliverers**). Some individuals expressed that their patients may have concerns about new methods, such as fears that vaginal rings could “disappear” inside the body, postpartum women feeling unready for intravaginal options, and pregnant women finding vaginal ring insertion uncomfortable (CFIR construct: **innovation recipients**). Community health providers (CHPs) and youth groups were suggested as effective channels for PrEP education (CFIR construct: **implementation facilitators**).

*“Through the CHPs [community health providers] in the community and even in the community meeting where HCW can be invited to give that information [about LA-PrEP] to different groups of people” – Female*,* healthcare planner*,* CAB*

### Implementation process

HCWs stressed the importance of assessing the needs of pregnant and postpartum women to recommend the most suitable PrEP method (CFIR construct: **assessing needs: innovation recipients**).

### Characteristics of systems and the intervention

CAB members highlighted the need for a reliable supply chain to ensure injectable PrEP and vaginal rings are readily available and accessible once the community is informed about these options (CFIR construct: **resource continuity**). Funding was also a concern, with external donors playing a key role in sustainability. There were fears that if partners (e.g. those who provide PrEP drugs to facilities) withdraw support, PrEP programs could collapse (CFIR constructs: **resource source**,** perceived sustainability**). Participants noted that some donors prioritize training for their own staff rather than facility-based HCWs, leaving gaps in knowledge and implementation capacity (CFIR construct: **external funding agent priorities**)

*“…as of now*,* if the partners pull out*,* you’ll start from zero*,* because the information will be gone with the partners who were…the staffs who were working with the partners.” – Female*,* healthcare planner*,* CAB*

*“even the partner when they come*,* they only concentrate on their particular staffs*,* so their trainings for their staffs everything that they are doing is that they are just concerned about their staff.” – Female*,* healthcare planner*,* CAB*

### Strategies to support LA-PrEP implementation

HCW and CAB discussions identified strategies across six ERIC clusters (Table 3). HCW and CAB discussions identified key strategies to support LA-PrEP implementation across multiple ERIC clusters. To enhance acceptability, participants suggested adapting LA-PrEP options to include pregnancy prevention, which could increase uptake within communities (ERIC strategy: **Promote adaptability**). Infrastructure changes were also emphasized, with HCWs and CAB members identifying alternative service sites, such as family planning clinics, that have the necessary setup for administering injections. Additionally, expanding access points to trusted community spaces, like pharmacies, was seen as a way to improve uptake (ERIC strategy: **Change service sites**). Stakeholder engagement was another crucial factor, with government workers, community health providers (CHPs), and teachers identified as key opinion leaders in promoting LA-PrEP (ERIC strategy: **Involve executive boards**). While CHPs play a critical role in disseminating information at the household level, their heavy workloads necessitate targeted sensitization efforts to ensure effective outreach (ERIC strategy: **Identify and prepare champions**). Other trusted community figures, including teachers, youth groups, and churches, were also recognized as important avenues for raising awareness.

To further engage consumers, participants recommended a multi-faceted approach to increasing awareness, including door-to-door outreach, information placement in pharmacies, and leveraging mass media (ERIC strategy: **Increase demand**,** use mass media**). However, they stressed that initial communication should come from trusted local sources, such as village elders and CHPs, before mass media campaigns are launched. Additionally, pregnant women were suggested as key advocates to encourage their peers to use PrEP during pregnancy (ERIC strategy: **Involve patients/consumers and family members**). Training for HCWs was highlighted as a recurring need, with participants emphasizing that providers require continuous education to confidently counsel patients on multiple PrEP options (ERIC strategy: **Conduct ongoing training**). CAB members also stressed the importance of training all facility providers to foster a collaborative learning environment (ERIC strategy: **Create a learning collaborative**). Finally, financial constraints were identified as a major barrier, with transportation reimbursements proposed as a way to encourage clinic visits for refills and testing (ERIC strategy: **Alter patient/consumer fees**).

**Table 3:** Implementation strategies for integration of LA-PrEP into reproductive healthcare settings identified by HCW and CAB members, with associated ERIC taxonomy

ERIC STRATEGY	SUPPORTING QUOTATIONS
**Adapt and Tailor to Context**	
Promote adaptability	*“I have really given one of the recommendations*,* that if we can develop a ring that at least is not mono in use can be dual*,* yes*,* that may be accepted in the community*,* that may increase acceptability.”* — Male, health system administrator, CAB
**Change infrastructure**	
Change service sites	*“Because of the setup which we have*,* that [family planning department] is the only available space*,* but remember she cannot inject in the clinician room*,* but it can be done in the area.”* — HCW, ID unknown
	*“The access points should be made easier within the community and at a place that the community have confidence [such as the] community pharmacy.”* — Male, health system administrator, CAB
**Develop stakeholder interrelationships**	
Identify and prepare champions	*“Because these services are being given at the facility*,* maybe in the facilities and according to researchers*,* the CHPs attached to these facilities need to be sensitized*,* even if it’s two or three days of sensitization and they are aware that if they pass the information to the household level.”* — Female, county government leader, CAB
	*“CHPs are the ones leading the households*,* however much they have a lot to do.”* — Female, county government leader, CAB
	*“Number one*,* using opinion leaders and key people in the society not leaving out headmasters and teachers because they offer very key populations in high school.”* — Male, healthcare planner, CAB
	*“We have the youth groups are a very good avenue for reaching the youths*,* and then they can get the information… And again*,* we can also use an organization like churches. Yeah*,* we can use churches to inform the people of the new techniques that we have at times.”* — Male, county government leader, CAB
**Engage consumers**	
Increase demand, use mass media	*“Door to door and use of pharmacy in the community that they know. You go to buy those painkillers*,* you find the leaflets hanged around there.”* —Female, PrEP ambassador, CAB
	*“The main issue there is just creating awareness and getting them informed. Through road shows and also the chiefs in the barazas.”* — Female, PrEP ambassador, CAB
	*“It is not everybody that goes to the hospital when they are sick. When they feel sick or feverish*,* they go and buy over-the-counter drugs. Believe me*,* when you put those leaflets in the pharmacy*,* the information can be passed very easily.”* — Male, community health volunteer, CAB
	*“Before using mass media*,* it should find that a physical person that has gone on the ground to tell the community the new drug which is coming. Because people might ask that why is that we are only hearing it on the radio and nobody has told us yet we have the village elders and the CHVs. We should have our CHVs proactive towards giving the information*,* before we are back on the mass media.”* — Male, community leader, CAB
Involve patients/ consumers and family members	*“Facilities should use pregnant women also as examples to encourage other women that taking PrEP during pregnancy is also safe.”* — Female, PrEP ambassador, CAB
**Train and educate stakeholders**	
Conduct ongoing training	*“I will comfortably be providing the information but still we need additional training to keep us more up to date. Things change here and there and us providers we need to be top of it all to give the correct information every time.”* — Male, pharmacy tech, HCW
Create a learning collaborative	*“The other staff should also be given the information so that it not left to us alone. It should be for the facility.”* — Male, clinical officer, HCW
Distribute educational materials	*“The government should come up with innovation… let them come up with the small gadget like this one [mWACh PrEP]*,* the one that serves with health issues*,* where be the government will employ people and to give information*,* health education on this one*,* recordings etc.*,* and then they send to the family or household and then they are supposed to be free.”* — Male, healthcare planner, CAB
**Utilize financial strategies**	
Alter patient/ consumer fees	*“[Male*,* community leader:] …So we realize that the financial support can also enhance them to come to the clinic for refill or testing because they know they can get the transport back home. * *[Female*,* PrEP ambassador:] Just to add on what [they] have said. The issue is financial issue about transport*,* once the patients are promised a token as transport. They can be eager to go and take PrEP which is for their benefit. As time goes by they will see the benefit of the drug and forget about the transport.”* — CAB

### Strategy prioritization

HCW discussions identified key tools, resources, and support needed to implement LA-PrEP in their settings, with common themes including training, space, commodities, and guidelines. However, the prioritization of these elements varied across HCW groups for “Ease of implementation” and “Most helpful” (Table 4). One focus group did not rank resources in terms of “Most helpful” due to time constraints. The number and type of prioritized strategies varied across groups, reflecting differences in rankings and context-specific perspectives. Differences in ranking order also reflected varying needs based on local contexts, with training and personnel consistently highlighted, alongside logistical considerations such as space and commodities.

**Table 4:** Prioritization activity

HCW group 1	HCW group 2	HCW group 3	HCW group 4
**Ease of implementation**			
1. Training/ sensitization/ knowledge	1. Space	1. Guidelines (providing its available)	1. Training (LA-PrEP), supported by guideline
2. Provision of tools	2. Personnel	2. Training	2. Commodities, tools
3. Personnel	3. Training	3. Personnel	3. Community sensitization
4. Guidelines (national level) – not easy to implement but it is key	4. IEC materials	4. Space	
		5. Commodities	
**Most helpful**			
1. Training	1.Commodities	1. Personnel	Not completed
2. Guidelines	2. Training	2. Training	
3. Personnel	3. Space	3. Commodities	
4. Commodities (syringes, gloves, needles)		4. Space	
5. Telecommunication (registers, tools for accountability)			

## Discussion

In our qualitative exploration grounded in the CFIR and ERIC frameworks, HCW and CAB members in Kenya provided insights into determinants and strategies for LA-PrEP implementation in reproductive healthcare settings. Determinants for the implementation of LA-PrEP into ANC and FP settings in Kenya, such as training, funding, and personnel, are intrinsically interdependent. Some strategies clearly addressed barriers (e.g. **conducting ongoing training** to support **access to knowledge/information**). However, some barriers had no clear solution, such as drug availability of long-acting options at clinics and access to funds to conduct training and community sensitization. As found in the prioritization activity, strategies for LA-PrEP implementation may vary by location in terms of helpfulness and ease, based on existing resources and infrastructure. The wide range of strategies identified, such as financial incentives for transportation, using CHPs to conduct community sensitization, and integrating LA-PrEP into FP clinics, hint at the multi-pronged approach needed to support implementation of LA-PrEP in this setting. Existing evidence indicates that both DVR and CAB-LA are safe for use during pregnancy and lactation; however, ongoing studies continue to evaluate their safety profiles [18, 34]. Suggested implementation determinants and strategies identified in this study provide a foundation for further exploration of LA-PrEP implementation in ANC, including assessments of health system readiness and evaluations of implementation outcomes.

Many of the implementation barriers identified in these FGDs are long-standing barriers for daily oral PrEP, but several are unique considerations for LA-PrEP methods. Barriers such as workforce shortages, limited time to address topics during visits, and challenges with drug availability are well-documented in the implementation of oral PrEP [21, 35–37] and may be exacerbated with the introduction of new LA-PrEP methods [38]. In our study, HCW expressed concerns around patient acceptance of DVR related to ring insertion, especially for pregnant and postpartum women. Existing evidence shows that DVR is safe and acceptable for pregnant women, and initial concerns are often alleviated with study staff support and increased product use experience [34, 39, 40]. The concerns highlighted by HCW in this study emphasize the need for patient education and provider training about DVR insertion and safety for use during pregnancy and breastfeeding. Conversely, HCW expressed confidence in their ability to administer injections for CAB-LA, drawing on their experience and familiarity with providing injectable contraceptives as a comparable skill. Similarly, studies on LA-PrEP acceptability among pregnant and postpartum women report high acceptance of injectable PrEP [41, 42]. Many FGD participants highlighted the appeal of LA-PrEP options for their discreet nature and their potential to reduce the stigma associated with daily pill-taking. However, LA-PrEP is not entirely free from stigma-related challenges. Previous studies of CAB-LA implementation have noted potential stigmatization due to healthcare workers’ moral judgments regarding the sexual behaviors of individuals using injectable PrEP [43], similar to themes found in our study. Additionally, if injections are administered in HIV clinics, they may still carry stigma due to their association with HIV-related care [37].

Additionally, it is also important to consider implementation barriers which did not come up in these FGDs but may still play an important role. Previous studies have noted that engagement with facility leadership as essential for overcoming challenges related to PrEP adoption and feasibility [35], but this theme did not appear in our study. HCW in our study expressed concerns about increased workloads associated with PrEP counseling, ring insertion, and administering injectables but there are other potential factors that could further complicate workloads, such as the need for additional HIV testing for CAB-LA monitoring [35, 37]. Future studies can conduct quantitative evaluation of clinics’ service availability and readiness to track longitudinal implementation of the introduction of LA-PrEP options [13]. ANC systems can be evaluated for readiness in terms of the WHO Building Blocks of service delivery, health workforce, health information systems, access to essential medicines, financing, and leadership/governance to support LA-PrEP in their settings [44], areas which were explicitly mentioned as implementation barriers in this study.

In our study, HCW and CAB participants identified barriers and strategies that mirror those observed in other reproductive health areas, such as family planning. For example, HCWs highlighted drug availability and stock-outs as key implementation challenges, along with the need for ongoing training to remain current on emerging PrEP options. Evidence from family planning similarly demonstrates that product availability can shape contraceptive method choice and patterns of method switching [45]. While not directly assessed in our study, these insights offer important considerations for the delivery of LA-PrEP, particularly as providers introduce and counsel on new prevention options. However, training on new PrEP methods alone is unlikely to be sufficient, as participants in our study highlighted the interconnected nature of implementation determinants. CHPs and other key opinion leaders, like teachers and youth groups, play key roles in disseminating health information, as evidenced in maternal health and family planning initiatives [46]. Strategies for engaging consumers, such as using trusted peers, community sources and mass media, can build on approaches successfully used for oral PrEP knowledge campaigns [47, 48]. Additionally, financial strategies, like transport reimbursement for clinic visits, could also be adapted from other health programs to enhance uptake [49, 50], and evidence from pregnancy and postpartum care settings suggests that financial incentives can improve engagement and retention in care during this period [51, 52]. These approaches collectively highlight opportunities for applying known strategies to integrate LA-PrEP into Kenyan ANC settings effectively and future studies should evaluate when, ow, and how many strategies should be employed in this setting.

Building on the findings of this research, we have identified several next steps to support the integration of LA-PrEP into ANC settings. Both HCWs and CAB members highlighted the importance of offering patients a choice of PrEP options that best suit their individual needs. Ensuring this level of patient-centered care requires health systems to proactively plan and forecast resource needs to support the availability of diverse PrEP methods. Accurate forecasting of resource needs for commodities and services requires a clear understanding of client preferences, which are not always available. Lessons from the FP sector underscore the value of integrating client perspectives early in the planning process [53]. Stated preference methods such as discrete choice experiments can help generate actionable insights to guide resource allocation and inform demand projections, ensuring that patient preferences are adequately considered in health system planning [41]. In addition, future research should test the strategies proposed to address these barriers, as well as probing for additional barriers that may not have been captured in the current analysis. Explicitly connecting the implementation barriers and strategies will be essential for designing effective packages to support the implementation of LA-PrEP. Moreover, systematically evaluating the direction and strength of influence of each determinant can help researchers prioritize those factors with the most significant impact on implementation outcomes [27].

This analysis has several limitations. First, the provider-identified strategies for implementing LA-PrEP may not align with how clients prefer to access information or services. Effective implementation strategies should incorporate both provider and client perspectives to ensure they address the needs and preferences of end-users. Second, some determinants of LA-PrEP implementation may be challenging to forecast without real-world experience in delivering these interventions, potentially limiting the predictive accuracy of our findings. As a result, many of the identified determinants and strategies were not specific to MCH contexts, including pregnancy and postpartum periods. Given the unique healthcare needs and challenges faced during these stages, this lack of specificity may limit the applicability of the findings to the priority populations. Nevertheless, the HCW and CAB participants possess significant knowledge and experience with oral PrEP implementation. Pre-implementation studies, such as this one, provide valuable insights that can accelerate the translation of research into practice once LA-PrEP becomes available. Furthermore, these data were collected prior to the publication of LEN results, a promising injectable PrEP with six-month administration. While some implementation determinants and strategies may be shared across CAB-LA and LEN, there may be important differences which are not captured here. Third, the HCW participants were specifically selected based on their active involvement in PrEP delivery or antenatal care. This purposive sampling approach, while valuable for gathering expert insights, may have inadvertently excluded perspectives from clinics with lower levels of PrEP integration. Consequently, the barriers to LA-PrEP delivery identified in this study may not capture the full range of challenges faced by facilities with less established PrEP programs. Furthermore, interviews did not explicitly probe clinical screening, diagnostic, or assessment processes, which limits insight into the full PrEP initiation cascade. Future work should incorporate cascade analyses or full workflow assessments to more comprehensively examine screening, diagnostic, and clinical processes supporting PrEP initiation. Similarly, the CAB members involved in this analysis were connected to studies related to PrEP use in pregnancy. Their association with these research efforts may have introduced a bias toward greater optimism regarding the acceptability and feasibility of LA-PrEP. As a result, there may be additional barriers to LA-PrEP implementation not identified in this study. By leveraging these preliminary findings, future work can address critical knowledge gaps and optimize the rollout of LA-PrEP in diverse healthcare settings.

## Conclusion

HCWs and CAB members in Kenya described a complex network of implementation determinants such as training, funding, and community sensitization, with several strategies addressing barriers such as access to knowledge and information. However, unresolved barriers, like drug availability and funding sources, remain significant obstacles. Future research should focus on refining and prioritizing implementation strategies, exploring additional barriers, and integrating client perspectives to ensure that LA-PrEP implementation is both effective and aligned with the needs of the pregnant and postpartum women. These findings also provide an initial evidence base to inform future programmatic planning and policy considerations as LA-PrEP is introduced in ANC settings.

### Contributions to the literature

- Determinants for the implementation of long-acting (LA-) PrEP into settings that pregnant and postpartum women access (e.g. antenatal care (ANC) and family planning [FP]) in Kenya, such as training, funding, and personnel, are intrinsically interdependent.
- There are essential facilitators to consider for LA-PrEP implementation, such as high healthcare worker motivation to provide multiple options and parallels between injectable contraceptives and injectable PrEP.
- Healthcare workers strongly endorsed ongoing training as an implementation strategy to address the concern around rapidly changing information related to LA-PrEP.
- Immediate integration of LA-PrEP into ANC or FP settings in Kenya can ensure implementation success and improve uptake of LA-PrEP among pregnant and postpartum women.

## Supplementary Information

Below is the link to the electronic supplementary material.

Supplementary Material

## Data Availability

The datasets generated and/or analyzed during the current study are not publicly available due to institutional data sharing policies and restrictions but may available from the corresponding author on reasonable request.

## Abbreviations

ANC: Antenatal care
CAB: Community advisory board
CCC: Comprehensive care clinic
CAB-LA: Long-acting cabotegravir
CFIR: Consolidated framework for implementation research
CHP: Community health provider
DVR: Dapivirine vaginal ring
EPIS: Exploration, Preparation, Implementation, Sustainment
ERIC: Expert recommendations for implementing change
FGD: Focus group discussion
FP: Family planning
HCW: Healthcare worker
LA-PrEP: Long-acting pre-exposure prophylaxis
LEN: Lenacapavir
MCH: Maternal and child health
