# Supplementary material for: A rapid qualitative analysis of health care worker and community focus groups on determinants and implementation strategies for long-acting PrEP in antenatal and postpartum settings in Kenya
Source: Discov Health Syst. 2026 Aug 26;5(1):74. doi: 10.1007/s44250-026-00415-x (PMC13518471; doi:10.1007/s44250-026-00415-x)
Supplement: Supplementary file 1 [file 44250_2026_415_MOESM1_ESM.docx]

**Appendix 1. PARQA checklist**

| Item number |  | Guidelines to support rigor and validity | Manuscript location |
| --- | --- | --- | --- |
| 1) | Rigorous Design | Articulate the research question and finite purpose of the project | Introduction  Page 7 |
| 2) |  | Describe the rationale for using rapid qualitative methods | Methods: Data management and analysis  Page 9 |
| 3) |  | Define what is meant by “rapid qualitative analysis.” | Methods: Data management and analysis  Page 10 |
| 4) |  | Consider whether a theory, model, or framework will be used to inform the study and if so, why and how | Methods: Data collection  Page 8 |
| 5) |  | Define the intended timeframe of data collection, analysis, and products/deliverables | Methods: Data collection  Page 8 |
| 6) |  | Plan for appropriate staffing | Methods: Data collection  Page 8 |
| 7) |  | Explain the purpose and timeline of the study, communication plan, and roles to all team members | Methods: Data collection  Page 8 |
| 8) | Data Collection | Develop and refine data collection tools to address specified research questions | Methods: Data collection  Page 8 |
| 9) |  | Pilot data collection instruments to ensure they are clear, feasible, and appropriately targeted | Methods: Data collection  Page 8 |
| 10) |  | Develop a plan for review throughout the data collection process | Methods: Data management and analysis  Page 10-11 |
| 11) | RQA: Summary Template Development | Develop and pilot test a user-friendly summary template | Methods: Data management and analysis  Page 11 |
| 12) |  | Ensure there are cross-references to raw data to support continuous comparison and validation necessary for RQA | Methods: Data management and analysis  Page 10 |
| 13) |  | Develop summaries that are accurate and concise, but detailed enough to meet project aims | Methods: Data management and analysis  Page 10 |
| 14) |  | Identify training and calibration processes to ensure consistency and accuracy in summaries | Methods: Data management and analysis  Page 10 |
| 15) | RQA: Matrix Analysis | Plan qualitative matrix structure to reflect project aims/ questions | Methods: Data management and analysis  Page 10 |
| 16) |  | Describe use of software for matrix analysis (e.g., Excel, Word) | Methods: Data management and analysis  Page 10 |
| 17) |  | Develop a plan for review throughout matrix analysis | Methods: Data management and analysis  Page 10 |
| 18) | Rapid Qualitative Data Synthesis | Conduct synthesis that is rigorous and responsive to research priorities | Results  Pages 11-17 |

**Appendix 2. Representational Statement/Declaration**

This study was informed by input from a Community Advisory Board (CAB) comprised of local community leaders, women from the community, representatives from the Kenyan Ministry of Health, and county-level health officials from maternal and child health (MCH) departments. The CAB was intentionally structured to provide broad community and health system oversight, rather than to be demographically representative of the general population or of specific service user groups.

CAB members contributed to the review of study objectives, methods, and procedures to ensure cultural and contextual appropriateness, supported ethical and implementation oversight, and provided input on the feasibility and acceptability of study activities within routine health system settings. As such, their perspectives primarily reflect community leadership and health system viewpoints, which are critical for informing implementation strategies.

We acknowledge that the CAB composition may not proportionately reflect the perspectives of younger populations, particularly in the context of Kenya’s predominantly youthful demographic. Consequently, findings informed by CAB input may emphasize system-level and leadership perspectives over those of adolescents and young adults. These perspectives are nonetheless essential for understanding the practical considerations of integrating interventions within existing health systems, and should be interpreted alongside research that directly centers the experiences of younger populations and service users.

**Appendix 3: Mapping of Interview Guide Questions to Rapid Qualitative Analysis Domains and CFIR Constructs**

## *Healthcare Workers (HCW)*

| **Question** | **Domain** | **CFIR constructs** |
| --- | --- | --- |
| How do you think pregnant and postpartum women would like a long-acting PrEP method?  Would they choose it over daily oral PrEP?  Which option would they prefer?  Would it help women with adherence, or would adherence still be an issue? | Benefits of multiple PrEP options  Challenges of multiple PrEP options | - Innovation Relative Advantage - Innovation Adaptability - Community characteristics (CFIR in LMIC) |
| How would adding another PrEP formulation make your job easier or harder? | Benefits of multiple PrEP options  Challenges of multiple PrEP options | - Available Resources - Compatibility |
| If pregnant women were offered a choice between three PrEP options, would you feel comfortable helping them make a choice that’s right for them? | Clinic resources | - Local Attitudes - Self-efficacy |
| Tell me about support and resources you currently have at this clinic to provide another type of PrEP? | Clinic resources | - Local Conditions - Available Resources - Team characteristics (CFIR in LMIC) - Resource source (CFIR in LMIC) - Resource continuity (CFIR in LMIC) |
| What information, training, or support would you need to feel comfortable offering a long-acting form of PrEP to pregnant and postpartum women?  Do you feel comfortable with both an injectable PrEP and vaginal ring?  Are there any considerations for offering an injectable vs a vaginal ring?  How would training or support look for an injectable vs a vaginal ring? | Integration strategies  Clinic resources | - Self-efficacy - Access to Knowledge & Information - Innovation Relative Advantage - Innovation Design - Innovation Complexity - Available Resources - Access to Knowledge & Information |
| Which of your suggested ideas would be the easiest to implement or do in your clinic? | Integration strategies | - Compatibility - Planning - Collective efficacy (CFIR in LMIC) |
| Which of your suggested ideas would be the most helpful to make you feel comfortable offering a long-acting PrEP in your clinic? | Integration strategies | - CFIR - Available Resources - Access to Knowledge & Information - Planning - Collective efficacy (CFIR in LMIC) |

## *Community Advisory Board (CAB)*

| **Question** | **Domain** | **Mapped domains & constructs** |
| --- | --- | --- |
| Think about the benefits and challenges of having multiple PrEP options available for HIV prevention. [***Give them time to think***] What benefits and challenges do you see for having multiple PrEP options available to pregnant and postpartum women specifically. What are they? | Benefits of multiple PrEP options  Challenges of multiple PrEP options | - Innovation Relative Advantage - Innovation Design - Complexity - Innovation Adaptability |
| Are there different benefits for pregnancy vs postpartum? | Benefits of multiple PrEP options  Challenges of multiple PrEP options | - Innovation Relative Advantage - Innovation Adaptability - Patient Needs & Resources |
| Are there any considerations for offering an injectable vs a vaginal ring? | Benefits of multiple PrEP options  Challenges of multiple PrEP options | - Innovation Relative Advantage - Innovation Design - Complexity |
| What suggestions do you have to make sure that this new PrEP formulations are acceptable and easy to access/practical for your community? | Acceptability | - Patient Needs & Resources - Partnerships & Connections - Perceived Scalability (CFIR in LMIC) |
| What do you think are the best strategies for sharing information about new PrEP products in your community? | Integration strategies | - Planning - Engaging |
| If pregnant women were offered a choice between three PrEP options, what tools/information would be useful to help them make their decision? | Integration strategies | - Patient Needs & Resources - Engaging - Access to Knowledge & Information |
